# 3D Bioprinted Human Cortical Neural Constructs Derived from Induced Pluripotent Stem Cells

**DOI:** 10.3390/jcm8101595

**Published:** 2019-10-02

**Authors:** Federico Salaris, Cristina Colosi, Carlo Brighi, Alessandro Soloperto, Valeria de Turris, Maria Cristina Benedetti, Silvia Ghirga, Maria Rosito, Silvia Di Angelantonio, Alessandro Rosa

**Affiliations:** 1 Center for Life Nano Science, Istituto Italiano di Tecnologia, Viale Regina Elena 291, 00161 Rome, Italy; federico.salaris@uniroma1.it (F.S.); cristinacolosi@gmail.com (C.C.); carlo.brighi@uniroma1.it (C.B.); alessandro.soloperto@iit.it (A.S.); valeria.deturris@iit.it (V.d.T.); silvia.ghirga@uniroma1.it (S.G.); maria.rosito@iit.it (M.R.); 2 Department of Biology and Biotechnology Charles Darwin, Sapienza University of Rome, P.le A. Moro 5, 00185 Rome, Italy; benedetti.1690350@studenti.uniroma1.it; 3 Department of Physiology and Pharmacology, Sapienza University of Rome, P.le A. Moro 5, 00185 Rome, Italy

**Keywords:** 3D bioprinting, biofabrication, 3D cultures, iPSCs, cortical neurons, calcium imaging, patch clamp

## Abstract

Bioprinting techniques use bioinks made of biocompatible non-living materials and cells to build 3D constructs in a controlled manner and with micrometric resolution. 3D bioprinted structures representative of several human tissues have been recently produced using cells derived by differentiation of induced pluripotent stem cells (iPSCs). Human iPSCs can be differentiated in a wide range of neurons and glia, providing an ideal tool for modeling the human nervous system. Here we report a neural construct generated by 3D bioprinting of cortical neurons and glial precursors derived from human iPSCs. We show that the extrusion-based printing process does not impair cell viability in the short and long term. Bioprinted cells can be further differentiated within the construct and properly express neuronal and astrocytic markers. Functional analysis of 3D bioprinted cells highlights an early stage of maturation and the establishment of early network activity behaviors. This work lays the basis for generating more complex and faithful 3D models of the human nervous systems by bioprinting neural cells derived from iPSCs.

## 1. Introduction

In three-dimensional (3D) bioprinting, cells and biocompatible materials are used as a biological ink (bioink) that can be organized in the 3D space with the goal of generating constructs mimicking organs and tissues. Recent advancements in biofabrication techniques have opened the possibility to apply 3D bioprinting methodologies to human pluripotent stem cells (hPSCs), including embryonic stem cells (ESCs) and induced pluripotent stem cells (iPSCs). The interest in using hPSCs as building blocks in 3D bioprinting comes from their ability to generate ideally any cell type of interest by in vitro differentiation. Laser-assisted [1] and extrusion-based [2] bioprinting are two layer-by-layer deposition methods recently applied to undifferentiated hPSCs [3,4,5,6]. Once embedded in a 3D construct, hPSCs maintain their plurilineage potential [3,4,5] and could be converted by directed differentiation into neural [4], cartilage [6] or cardiac cells [3]. An alternative approach relies on prior differentiation of hPSCs into cell types of interest, which are then printed to generate tissue-like 3D constructs. Examples of liver [7,8,9], cardiac [9,10], vascular [11], cornea [12] and spinal cord [13,14] cells, all derived from hPSCs by conventional differentiation and subsequently used for bioprinting, have been recently reported. Notably, to the best of our knowledge, cortical neurons and glial cells derived from hPSCs have never been successfully used for bioprinting. Obtaining cells that cannot be isolated from primary human tissues is one of the major purposes of hPSCs, which have been successfully used to generate a wide variety of derivatives of the nervous system, including neuron subtypes of interest for translational or basic science applications [15].

In this work we took advantage of a custom-made extrusion-based bioprinter, implemented with co-axial wet-spinning microfluidic devices [16], to build 3D constructs made of iPSC-derived cortical neurons and glial cells. Optimization of the printing process and bioink composition resulted in high survival of human neural cells. Bioprinting did not impair further differentiation of the cells within the 3D construct. We also report long term maintenance and acquisition of mature functional properties.

## 2. Experimental Section

### 2.1. Cell Culture and Differentiation

Generation and maintenance of iPSCs (WT I line) is described in Lenzi et al. (2015) [17]. In brief, cells were cultured in Nutristem-XF (Biological Industries, Cromwell, CT, USA) supplemented with 0.1% Penicillin-Streptomycin (Thermo Fisher Scientific, Waltham, MA, USA) in hESC-qualified Matrigel (CORNING, New York, NY, USA) coated plates. Medium was changed every day and cells were passaged every 4–5 days using 1 mg/mL Dispase (Gibco, Waltham, MA, USA). The cortical neurons differentiation protocol has been adapted and modified from Shi et al. (2012) [18]. iPSCs were treated with Accutase (Thermo Fisher Scientific) promoting single cell dissociation and plated in Matrigel coated dishes in Nutristem-XF supplemented with 10 µM Rock Inhibitor (Enzo Life Sciences, Farmingdale, NY, USA) and 0.1% Penicillin-Streptomycin with a seeding density of 65,000 cells per cm^2^. After three days, medium was changed to N2B27 medium (DMEM-F12, Dulbecco’s Modified Eagle’s Medium/Nutrient Mixture F-12 Ham, Sigma Aldrich; Neurobasal Medium, Gibco; 1X N2 supplement, Thermo Fisher Scientific; 1X Glutamax, Thermo Fisher Scientific; 1X NEAA, Thermo Fisher Scientific; 1X B27, Miltenyi Biotech; 1X Penicillin-Streptomycin) supplemented with SMAD inhibitors, 10 µM SB431542 and 500 nM LDN-193189 (both from Cayman Chemical, Ann Arbor, MI, USA). This was considered day 0 (D0). Medium was changed every day. After 10 days, cells were passaged with 1 mg/mL Dispase and re-plated into poly-L-ornithine/laminin (Sigma Aldrich, St. Louis, MO, USA) coated dishes in N2B27 medium. Starting from day 10, medium was changed every other day. At day 20, cells were dissociated using Accutase and plated into poly-L-ornithine/laminin coated dishes with a seeding density of 65,000 cells per cm^2^ in N2B27 medium supplemented with 10 µM Rock Inhibitor for 24 h. Medium was changed twice a week and 2 µM Cyclopamine (Merck, Kenilworth, NJ, USA) was supplemented to N2B27 medium at day 27 for 4 d. Around day 30, cells were dissociated again with Accutase and re-plated into poly-L-ornithine/laminin coated dishes with a seeding density of 65,000 cells/cm^2^ in N2B27 medium supplemented with 10 µM Rock Inhibitor for 24 h. From day 40, N2B27 medium was supplemented with 20 ng/mL BDNF (Sigma Aldrich, St. Louis, MO, USA), 20 ng/mL GDNF (Peprotech, London, UK), 200 ng/mL Ascorbic Acid (Sigma Aldrich), 1 mM cyclic AMP (Sigma Aldrich, St. Louis, MO, USA) and 5 µM DAPT (Adipogen Life Sciences, San Diego, CA, USA).

### 2.2. Preparation of Gel-Adhesive Glass Substrates and Bioink for 3D Bioprinting

Standard microscopy glass slides were functionalized following a published protocol [19] with minor changes. Briefly, standard glass slides were exposed to air plasma (3 min, 27 W, 600 mTorr) and quickly soaked in a 5% v/v solution of 3-aminopropyl triethoxysilane (Aptes, Sigma Aldrich, St. Louis, MO, USA) in deionized water for 2 h, washed with deionized water and ethanol and then air dried. Afterwards, slides were soaked in 0.2 M solution of 4-morpholineethanesulfonic acid (MES, PH4.5, Sigma Aldrich, St. Louis, MO, USA) containing 1% w/v alginate (Fmc Biopolymers, Philadelphia, PA, USA), EDC (0.4% w/v; Sigma Aldrich, St. Louis, MO, USA) and NHS (0.3% w/v; Sigma Aldrich, St. Louis, MO, USA) overnight at room temperature. Finally, slides were washed with water and ethanol, air dried, cut into 5 mm × 5 mm squares using a glass cutting pen, UV-sterilized and stored for later use. Alginate solution was prepared by dissolving alginate powder (GP1740, Fmc Biopolymers, Philadelphia, PA, USA) in 25 mM HEPES buffered saline (HBS). A stock solution of alginate was prepared at a concentration of 4% w/v, sterile-filtered, divided in working aliquots and stored at +4 °C for later use. The day of the bioprinting experiment, Matrigel precursor solution was thawed in ice for 2 h and mixed with alginate stock solution at a ratio of 1:1 v/v. Typically, 300 µL of Matrigel/alginate mixture was prepared for each experiment. All solutions were manipulated and kept in ice baths. Differentiating cells were collected from cell culture plates by single cell dissociation mediated by Accutase treatment. Cells were resuspended in the Matrigel/alginate mixture at a 1:1 ratio to obtain the bioink with 2% alginate as final concentration.

### 2.3. 3D Cell Printing and Post-Processing of Printed Samples

**Figure 1:**
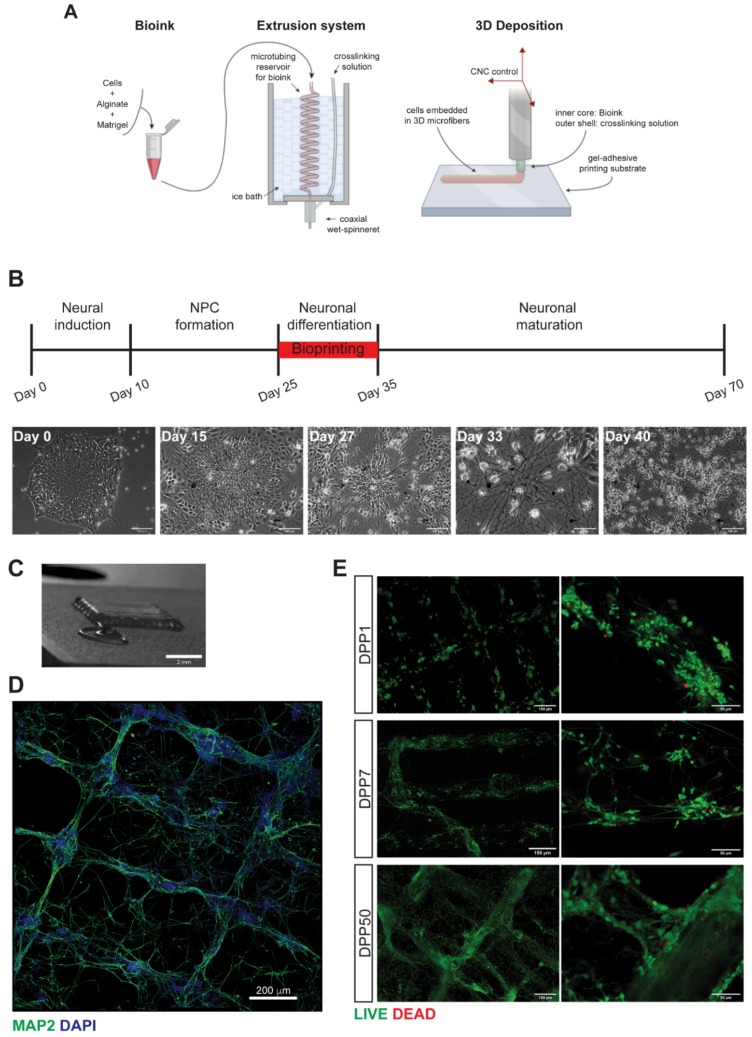
3D bioprinting method and analysis of viability post printing. (**A**) Schematic representation of the outline of the bioprinting method. (**B**) Outline of the human induced pluripotent stem cell (iPSC) neural differentiation protocol in conventional 2D culture and representative images of differentiating cells in these conditions at the indicated time points. The window of time in which cells have been dissociated for bioprinting experiments in this work is indicated in red. (**C**) Image of the printed 3D construct. Scalebar: 2 mm. (**D**) Mosaic reconstruction of confocal images of bioprinted neural cells at DPP7, stained with a MAP2 antibody (green) and DAPI (blue). Scalebar: 200 µm. (**E**) Live (green) and dead (red) cell staining in the bioprinted construct at the indicated days post printing (DPP). Scalebar: 150 µm (left panels); 50 µm (right panels).

The bioink was loaded in a reservoir consisting of a micro-tube coil of known internal volume, as schematized in Figure 1A. The reservoir was placed in a poly(methyl methacrylate) cylindrical tank (3 cm radius, 15 cm height) covered with a thermal isolating tape (Armaflex L414, Armacell, Munster, DE) and filled with ice in order to prevent the gelation of Matrigel in the reservoir. The total dimension of the system, shown in Figure S1, was rationalized in order to limit the encumbrance of the extruder while ensuring the maintenance of a temperature around 0 °C for the total duration of the printing step (~1 h). The reservoir was connected with the internal needle of a coaxial wet-spinning extruder, while the outer needle was fed with a calcium chloride solution (225 mM CaCl_2_ in HBS). Two independent microfluidic pumps (Cetoni, Korbussen, DE) controlled the flow of the bioink (5 µL/min) and the crosslinking solution (3.5 µL/min) through the coaxial extruder. The extruder and the ice-bath tank were mounted on a three-axis motorized system (PI-miCos, Eschbach, DE) with a computer-numerical-control (CNC) interface (Twintec, Auburn, WA, USA). Printing instructions were expressed in g-code language, and printing codes were generated using a custom MATLAB algorithm. The geometry described in these codes consisted of two alternating perpendicular layers of microfibers with theoretical diameter of 100 µm, separated by gaps of 200 µm, a layer thickness of 100 µm, deposited at a speed of 240 mm/min, forming a squared fiber mesh of 5 mm × 5 mm × 200 µm. Typically, printing time for generated codes was around 40–50 s, and each sample was constituted of 3 to 5 µL of solution depending on the desired dimension of the construct. Printed samples were collected, washed with sterile saline solution and placed in cell culture incubator for 10 min to trigger the gelation process of Matrigel. Samples were then transferred in 12-well cell culture plates, soaked with cell culture media and incubated for additional 2 h to terminate Matrigel gelation. Afterwards, samples were exposed to alginate-lyase enzyme (Sigma Aldrich, 0.2 µg/mL in cell culture media) overnight, washed with fresh media and maintained in culture for characterization and maturation.

### 2.4. RNA Analysis

Total RNA was extracted with the Quick RNA MiniPrep (Zymo Research, Freiburg, DE) and retrotranscribed with iScript Reverse Transcription Supermix for RT-qPCR (Bio-Rad, Hercules, CA, USA). Targets were analyzed by PCR with the enzyme MyTaq DNA Polymerase (Bioline, Boston, MA, USA). Thirty cycles of amplification were used for *PAX6* and *GAPDH*, while *FOXG1*, *TBR1*, *TBR2* and *GFAP* were amplified for 34 reaction cycles. The internal control used was the housekeeping gene *GAPDH*. Primer sequences are reported in Table S1.

### 2.5. Live/Dead Cell Analysis

Cell viability was assessed with the LIVE/DEAD Viability/Cytotoxicity Kit (Thermo Fisher Scientific), which uses green-fluorescent calcein AM and red-fluorescent ethidium homodimer-1 to identify live and dead cells, respectively, according to the manufacturer’s instructions. Briefly, bioprinted constructs were incubated with calcein-AM and ethidium homodimer-1 at 37 °C for 30 min, followed by a PBS wash, and a further media change before acquisition. Image acquisition was performed with a custom fluorescent integrated system (Crisel Instruments, Rome, IT) based on an IX73 Olympus inverted microscope equipped with the x-light spinning disk module (Crestoptics, Rome, IT) for confocal acquisition, Lumencor Spectra X LED illumination and a CoolSNAP MYO CCD camera (Photometrics, Tucson, AZ, USA). The widefield images were acquired using Metamorph software version 7.10.2 (Molecular Device, San Jose, CA, USA) with 10×, 20× and 40× air objectives. The construct was sectioned in z with a step size of 5 µm to obtain at least five optical planes per construct to capture the whole structure. For the live/dead cells quantification, the entire image stacks were analyzed in 3D and cells were counted using Spots in Imaris 8.1.2 (Bitplane, Belfast, UK); nine fields were analyzed for each time point. Percentage of viability is reported as the mean value ± standard deviation of the mean, from three independent bioprinted constructs at DPP1 and DPP7 and one bioprinted construct at DPP50.

### 2.6. Immunostaining

Cells were fixed in 4% paraformaldehyde for 15 min at room temperature and washed twice with PBS. Fixed cells were then permeabilized with PBS containing 0.2% Triton X-100 for 10 min at room temperature and incubated overnight with primary antibodies at 4 °C. The primary antibodies used were anti-PAX6 (1:50, sc-81649, Santa Cruz Biotechnology, Dallas, TX, USA), anti-NCAD (1:100, ab18203, Abcam, Cambridge, UK), anti-TUJ1 (1:1000, T2200, Sigma-Aldrich), anti-MAP2 (1:2000, ab5392, Abcam), anti-NeuN (1:50, MAB377, Merck Millipore), anti-GFAP (1:500, MAB360, Merck Millipore) and anti-TBR1 (1:150, 20932-1-AP, Proteintech, Rosemont, IL, USA). The secondary antibodies used were goat anti-mouse Alexa Fluor 488 (1:250, Immunological Sciences, Rome, IT), goat anti-chicken Alexa Fluor 488 (1:500, Thermo Fisher Scientific), goat anti-rabbit Alexa Fluor 488 (1:250, Immunological Sciences), goat anti-mouse Alexa Fluor 594 (1:500, Thermo Fisher Scientific), goat anti-rabbit Alexa Fluor 594 (1:500, Thermo Fisher Scientific) and goat anti-rabbit Alexa Fluor 647 (1:500, Thermo Fisher Scientific). DAPI (Sigma-Aldrich) was used to label nuclei.

### 2.7. Microscopy Imaging

Confocal images of panels 1D, 2D and 2D’ were acquired at the Olympus iX83 FluoView1200 laser scanning confocal microscope using an air 10× NA0.4 or a silicon oil 30× NA1.05 objective (Olympus, Shinjuku, JP) and 405, 473, 559 and 635 nm lasers. Filter setting for DAPI, Alexa Fluor 488, Alexa Fluor 594 and Alexa Fluor 647 were used when needed. Each stack consisted of individual images with a z-step of 0.5, 5 and 1 µm respectively. Stack images of 1024 × 1024 pixels were stitched together in a mosaic view with the Multi Area Viewer tool of Fluoview 4.2 image software (Olympus). The 3D rendering shown was performed using the Imaris image analysis software v.8.1.2 (Bitplane). Widefield images of panels 2C were acquired with the same system described above with a 20× air objective.

### 2.8. Patch Clamp Recordings

Whole-cell patch-clamp recordings were used for the functional characterization of 3D bioprinted constructs. Cells in the 3D bioprinted structures were visualized with a BX51WI microscope (OLYMPUS), in a recording chamber continuously perfused with an external solution containing 140 mM NaCl, 2.8 mM KCl, 2 mM CaCl_2_, 2 mM MgCl_2_, 10 mM HEPES and 10 mM D-glucose (pH 7.3 with NaOH; 290 mOsm) at room temperature. Borosilicate pipettes were filled with a solution containing 140 mM K-gluconate, 5 mM BAPTA, 2 mM MgCl_2_, 10 mM HEPES, 2 mM Mg-ATP and 0.3 mM Na-GTP (pH 7.3 with KOH; 280 mOsm). Voltage- and current-clamp recordings were performed using Axon DigiData 1550 (MOLECULAR DEVICES). Signals were filtered at 10 KHz, digitized (25 kHz) and collected using Clampex 10 (MOLECULAR DEVICES). Whole-cell capacitance (Cm), cell membrane resistance (Rm) and resting membrane potential (RMP) were measured on-line by Clampex. Cells were clamped at –70 and 0 mV to measure spontaneous activity. An on-line P4 leak subtraction protocol was used for all recordings of voltage-activated currents. Voltage steps (50 ms duration) from −80 to +40 mV (10 mV increment; holding potential −70 mV) were applied to study voltage-activated sodium currents. Voltage-activated potassium currents were evoked by voltage steps (50 ms duration) from −80 to +40 mV (10 mV increment; holding potential −70 mV). Firing properties were investigated in current-clamp mode, injecting current pulses (1 s duration) of increasing amplitude (from 10 to 80 pA; 10 pA increment), after imposing a membrane potential of −70 mV to each cell (injection of −79 ± 20 pA). Data were analyzed off-line with Clampfit 10 and Origin 7 software.

### 2.9. Calcium Imaging Recordings

Fluorescence images were acquired at room temperature using a customized digital imaging microscope. Between 5–8 field of views (FOVs) per bioprinted construct were recorded in each experiment session. Excitation of calcium dye was achieved using a 1-nm-bandwidth monocromator (Cairn Optoscan, Faversham, UK) equipped with a 150 W xenon lamp. Fluorescence was visualized using the upright microscope Olympus BX51WI equipped with a 40× water immersion objective and a CoolSnap Myo camera. Image acquisition and processing were obtained using MetaFluor software (Molecular Devices). Changes in the intracellular Ca^2+^ level were monitored using the high-affinity Ca^2+^-sensitive indicator Fluo4-AM (Invitrogen). 3D bioprinted constructs were loaded by incubating for 30 min at 37 °C in external solution containing the following: 140 mM NaCl, 2.8 mM KCl, 2 mM CaCl_2_, 2 mM MgCl_2_, 10 mM HEPES, 10 mM D-glucose (pH 7.3 with NaOH; 290 mOsm) plus 5 µM Fluo4-AM. A custom-made MATLAB guided user interface (GUI) was used to perform calcium imaging data analysis. Fluorescence data collected as a series of images were converted to three-dimensional MATLAB files and the neurons were manually selected from the time-averaged fluorescence recording before running the trace extraction and analysis. Tens of neurons were identified for each field scanned, depending on the confluence and seeding density of the cultures. The calcium traces were acquired with a sampling frequency of 2 Hz and signals were normalized as a function of ΔF/F_0_ = (F − F_0_)/F_0_, where F is the current fluorescence intensity at any time point and F_0_ is the basal fluorescence intensity. Single calcium events were detected on the basis of a previously published method [20]. Threshold for peak amplitude, initially set to 3% of the baseline value, was manually adjustable to improve the detection depending on the recording conditions (signal-to-noise ratio, acquisition rate, etc.). Results were visualized, and eventual false or missing detections were manually corrected. Raster plots were created to visualize asynchronous (appearing as sparse vertical lines) and synchronous (appearing as a series of vertically aligned lines) results and the linear dependence between each pair of neurons was calculated by means of Pearson correlation coefficient from binary traces. Neuron firing rate, amplitude of the events and synchrony of the network (evaluated as the relative number of simultaneous events) were exported in Microsoft Excel to perform statistics. Statistical analysis was performed in GraphPad Prism 7 or OriginPro 6.0.

## 3. Results

### 3.1. 3D Bioprinting of Differentiating Human iPSC-Derived Neurons and Glia

An outline of the 3D bioprinting method developed in this work is shown in Figure 1A. We used a customized extrusion bioprinter developed in-house (Figure S1). This platform consists of a custom extrusion 3D bioprinter integrating a microfluidic printing head constituted of two independent needles arranged in a coaxial configuration. The deposition strategy is based on the use of calcium-alginate gel as templating agent for the printing of blended extracellular matrices and cells. This provides a precise control on the relative position of cells within the 3D construct, down to the micrometer scale with high reproducibility [16], independently on the 3D embedding matrix of election. Reportedly, bioink composition is crucial to ensure long-term iPSCs viability and maintenance of 3D structures [3,6]. Pilot experiments revealed that Matrigel is the best candidate for in vitro differentiation of neuronal cells in 3D, when compared with transglutaminase/gelatin or photo-crosslinked gelatin methacryloyl gels. For 3D printing experiments, different ratios of Matrigel/alginate and post-printing treatments have been tested. We obtained the best results using a solution containing 2% w/v alginate and 0.5× Matrigel (~50% dilution from stock), printed using 0.33 mM CaCl_2_ crosslinking solution, and subsequently exposing the printed construct to alginate-lyase enzyme at a concentration of 0.2 µg/mL in cell culture media for 12 h, starting the exposure 3 h after the printing protocol.

The cellular components of the bioink, neuronal and glial precursors, were derived by differentiation of human iPSCs by a multistep protocol in conventional bidimensional (2D) culture conditions (Figure 1B). Efficient induction of a neural cortical fate was obtained by initial dual SMAD inhibition and subsequent block of Hedgehog signaling with cyclopamine [16]. Representative images of differentiating cells are shown in Figure S2. During this standard differentiation process, human iPSCs exited from pluripotency (loss of *NANOG* expression) and gradually acquired a neural character, as shown by the progressive expression of neural progenitor cells (NPCs; *PAX6*, *NCAD*), neuronal precursors (*TBR2*, *FOXG1*) and neurons (*TBR1*, *TUJ1*, *NeuN*, *MAP2*) markers (Figure S2A). Further characterization by immunostaining analysis showed progressive acquisition of a neuronal morphology and expression of neurofilament proteins (Figure S2B,C). The astrocyte marker *GFAP* was also expressed at late time points (Figure S2B,C).

### 3.2. Characterization of 3D Bioprinted Neural Constructs

Neural cells differentiated for about 4 weeks were dissociated, resuspended in the Matrigel/alginate solution and printed. We have performed several experiments in which cells were dissociated in the window of time between day 25 and day 35 of differentiation (indicated in red in the diagram of Figure 1B). During the printing process, the bioink and the crosslinking solution met at the ending tip of the coaxial extruder. Here, Ca^2+^ ions triggered the gelation of alginate in the bioink. This gel adhered to the functionalized glass substrate so that, by moving the extruder, a micrometric cell-embedding gel fiber was spun out and deposited in pre-determined positions. In this work we printed the cells as a reticulum (Figure 1C; Movies S1 and S2). Such architecture was chosen as it allows optimal perfusion of culture medium, which can reach all the cells in the construct. Moreover, areas with lower and higher cell densities are formed along the fibers and at the crossing points, respectively, providing useful information on the behavior of the cells in the 3D construct under different density conditions. Alginate removal by enzymatic treatment 3 h after the printing process promoted the acquisition of neuronal morphology by the first day post printing (Figure S3). Notably, such mild enzymatic treatment did not affect the shape of the printed construct, which was stabilized by Matrigel polymerization. Immunostaining of neurofilaments showed that the structure of the reticulum was maintained over time and that neuronal cells projected their axons and dendrites both within and across the fibers (Figure 1D). Printed cells were then analyzed in terms of viability at different days post printing (DPP). Results shown in Figure 1E indicated that the great majority of the cells were viable at DPP1 (78 ± 3.8% live cells; average ± standard deviation; three constructs, nine fields each) and DPP7 (71 ± 3.5% live cells; average ± standard deviation; three constructs, nine fields each), suggesting that both physical parameters and bioink formulation did not harm neural cells during and immediately after the printing process. Moreover, viability was consistently maintained over time as assessed by live/dead staining up to DPP50 (68 ± 8% live cells; average ± standard deviation; one construct, nine fields). We noticed that the reticulum structure was to some extent maintained at this late time point.

**Figure 2:**
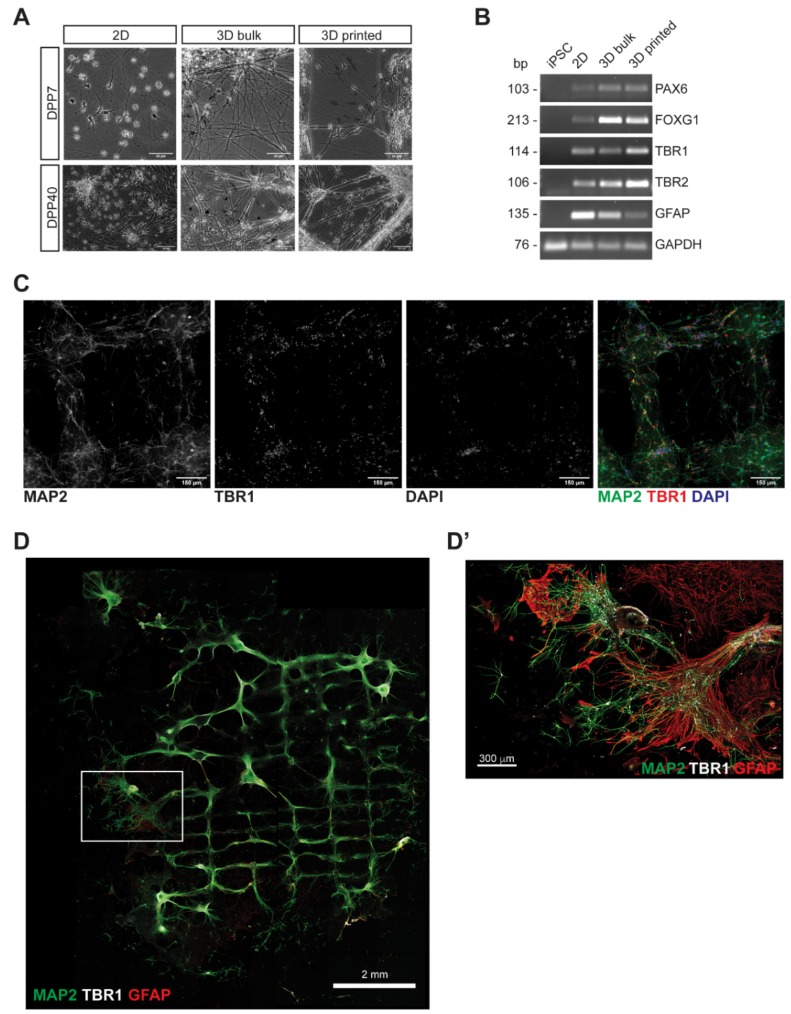
Analysis of neural marker expression in the 3D bioprinted construct. (**A**) Phase contrast images of cells within the 3D bioprinted construct (“3D printed” panels), at the indicated days post printing, and cells in conventional monolayer conditions (“2D” panels) or resuspended in the bioink (“3D bulk” panels) and maintained for the same time of differentiation. (**B**) RT-PCR analysis of neuronal progenitor markers (*PAX6*, *FOXG1*, *TBR2*), a cortical neuron marker (*TBR1*) and an astrocyte marker (*GFAP*). GAPDH was used as a housekeeping control. (**C**) Immunostaining analysis of bioprinted cells at DPP7. MAP2 (green), TBR1 (red) and DAPI (blue) signals are shown. Scalebar: 150 µm. (**D**) Mosaic reconstruction of confocal images of bioprinted neural cells at DPP70, showing the entire sample, stained with MAP2 (green), TBR1 (white) and GFAP (red) antibodies. Scalebar: 2 mm. (**D’**) Mosaic reconstruction of confocal images of the region inside the white box in panel D, acquired at higher resolution. Scalebar: 300 µm.

We then assessed possible alterations in neuronal cell fate acquisition caused by either the printing process and/or subsequent cell differentiation within the 3D bioprinted construct. Bioprinted cells were compared with cells maintained in conventional 2D conditions for the same time and cells that were encapsulated in bioink droplets not subjected to printing process (3D bulk). Neuronal morphology was maintained intact in both 3D bulk and 3D bioprinted cells at DPP7 and DPP40 (Figure 2A). In the same samples, marker analysis by RT-PCR showed proper expression of: *PAX6*, *FOXG1* and *TBR2* as neuronal progenitor markers; *TBR1*, which reveals the presence of mature cortical neurons; and *GFAP*, a common astrocyte marker (Figure 2B and Figure S4). These results were further supported by immunostaining analyses of TBR1 and MAP2 at DPP7 (Figure 2C). Bioprinted neural cells were maintained in neuronal differentiation medium up to DPP70. At this late time point the reticulum structure was, to some extent, maintained and cells properly expressed neuronal and astroglial markers (Figure 2D,D’).

Collectively, these results demonstrate that iPSC-derived cortical neuronal cells can be bioprinted and further cultured in 3D constructs without causing major survival and differentiation issues.

### 3.3. Functional Analysis

**Figure 3:**
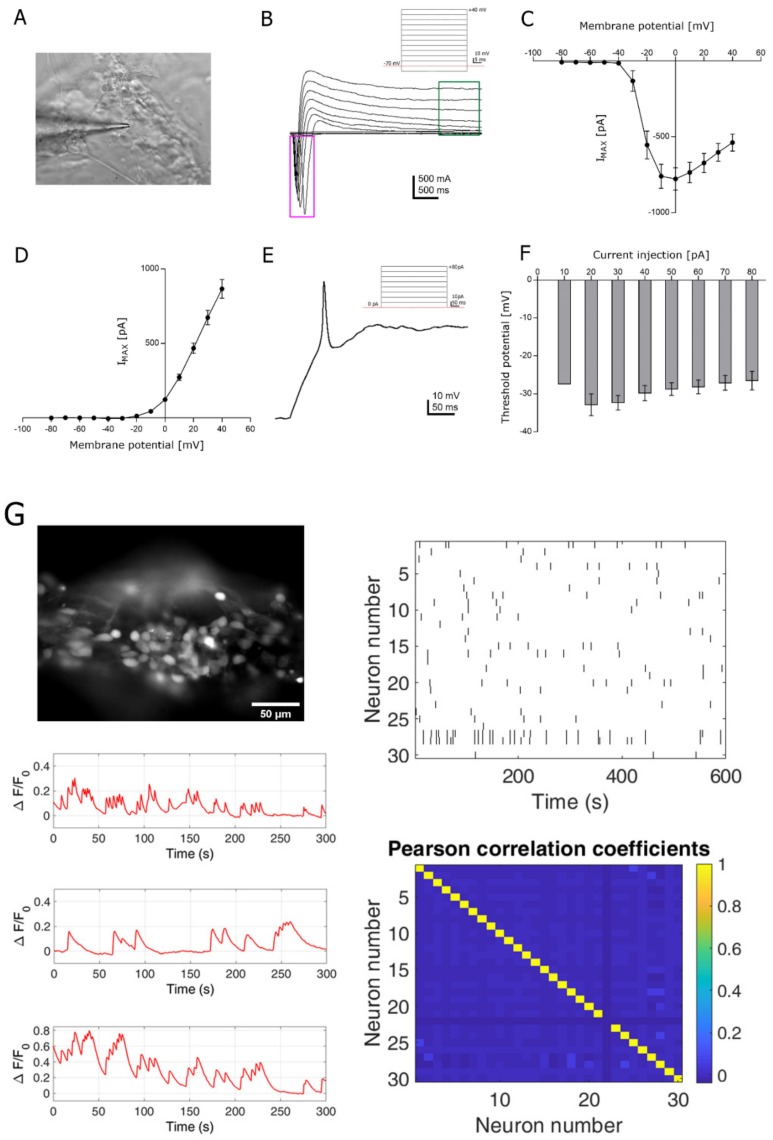
Functional analysis of the 3D bioprinted construct. (**A**) Single-cell patch-clamp recording of an iPSC-derived neuronal cell encapsulated in the 3D bioprinted construct at DPP7. (**B**) Representative scheme of the recording protocol is shown. The inward sodium currents are highlighted in the purple box and the permanent outward potassium currents are highlighted in the green box. (**C**) Average trace of the large inward voltage-dependent Na^+^ currents. (**D**) Average trace of the outward voltage-dependent K^+^ currents. (**E**) A single action potential evoked in current clamp recording is shown. The minimum current required to elicit firing was 10 pA, however more of the 50% of tested cells (*n* = 9 out of 15) responded to 20 pA (**F**). (**G**) Calcium traces as a function of ΔF/F_0_ of cortical neurons isolated within the 3D network shown on the left at DPP7. On the right, a representation of the firing pattern and a relative heatmap of the Pearson correlation coefficients within the cells of the same network are shown.

Single-cell patch-clamp and time-lapse calcium imaging recordings were then performed to assess the degree of maturation achieved by the 3D bioprinted construct. Even though the 3D construct was 300 µm thick, the selected bioink displayed sufficient transparency to visible light and softness to patch pipette insertion (Figure 3A). Using patch clamp recordings, we investigated the expression of the passive and active membrane properties on 3D bioprinted cortical neurons at day 7 after printing. As expected at this experimental point, resting membrane potential (−17.7 ± 1.5 mV; *n* = 36), cell capacitance (14.8 ± 0.89 pF; *n* = 45) and membrane resistance values (1.97 ± 0.23 MΩ; *n* = 44) were typical of neuronal progenitors [21] and similar to those observed in parallel 2D cultures (Figure S5), indicating that the printing process did not impair neuronal viability. We then characterized the ability of cortical neurons to generate action potentials. Neurons in the 3D construct displayed large inward voltage-dependent Na^+^ currents (−777.31 ± 73.16 pA at 0 mV; *n* = 43; Figure 3B,C and Figure S5) which activated near −40 mV and peaked at 0 mV, and voltage-dependent K+ currents (865.75 ± 63.28 pA at +40 mV; *n* = 43; Figure 3B,D and Figure S5). Current pulses were able to induce action potentials in almost all tested cells. The mean threshold for first action potential generation was −32.85 ± 2.86 mV (*n* = 15; 20 pA of current injection). However, the minimum current required to elicit firing in some of the tested cells was 10 pA (Figure 3E,F). As expected, no synaptic activity was recorded at 7 days post printing (data not shown). 

Given the optical transparency of the 3D bioprinted constructs at DPP7, fluorescence time-lapse recordings lasting 5 min each were performed, thus preserving a good signal-to-noise ratio (Figure 3G). Fluorescence time-lapse analysis of spontaneous calcium oscillation in Fluo4-AM loaded 3D neuronal network indicated the presence of individual calcium activity (mean firing frequency = 0.015 ± 0.001 Hz; FOVs = 38; mean firing amplitude = 0.083 ± 0.002 A.U.) with little synchronized firing (syncro index = 0.223 ± 0.020; FOVs = 38). The small degree of synchronous activity was confirmed by the low correlation coefficient value between each pair of neurons in the field as displayed by the heatmap in Figure 3G (average correlation coefficient value = 0.006; max correlation coefficient value = 0.046 ± 0.005; FOV = 38), thus indicating the establishment of early and immature neuronal networks.

## 4. Discussion

In this paper we describe a method to obtain 3D cortical constructs in which human cortical neurons and glial cells survive in the long term, holding their cellular characteristics and functional properties. Moving from conventional neuronal cultures, in 2D, to more realistic 3D models is considered a crucial advancement in neurobiology [22]. The recent discovery that differentiating hPSCs have the ability to self-assemble into brain organoids, which recapitulate to some extent the brain structure in 3D [23], has given a twist in the way neurodevelopmental and neurodegenerative diseases are modeled and approached [24]. 3D bioprinting could provide important advantages, in terms of automation and reproducibility, over self-assembled brain organoids [25]. Recent reports showed that undifferentiated human iPSCs and ESCs can be bioprinted and then converted, post-printing, into cell types of interest [4]. This approach will not likely generate useful artificial tissues, as it does not allow control on the position of individual cell types, generated during differentiation, within the construct. The complementary approach, used in this work and in [13,14], and consisting in bioprinting specific cell types obtained by pre-printing hPSCs differentiation, would be more advantageous, allowing better control of the resulting bioprinted construct. 

Bioprinting neurons and glial cells represents a challenge. Neurons are vulnerable cells in vitro and environmental stress due to the printing process may affect neural cell viability and influence further differentiation and maturation. Our work is the result of an extensive effort in the optimization of the bioprinting process and bioink composition, with the goal to define proper conditions for generating human artificial 3D cortical neural tissues from hiPSCs. The generation of a bioprinted constructs, by combining hiPSC-derived spinal neuronal progenitor cells and mouse oligodendrocyte progenitor cells, has been recently reported by Joung et al. [13]. Moreover, spinal cord neural progenitors from hiPSCs have been successfully bioprinted by using a commercial lab-on-a-printer platform [14,26]. Here, for the first time, we describe the generation of constructs made of cortical neurons and glial cells by a custom extrusion bioprinter. In this work, we have obtained the best results with a bioink made of Matrigel and alginate. The selection of the bioink most suitable for the viability of the 3D construct remains a controversial issue. Indeed, both fibrin-based and Matrigel-based bioinks have been previously used for bioprinting hiPSC-derived spinal neural cells [13,14]. Matrigel, which is a matrix preparation extracted from the Engelbreth-Holm-Swarm mouse sarcoma, had been successfully used as a bioink component for the generation of hiPSC-derived cardiac and spinal cord bioprinted constructs [3,13]. However, its composition is rather undefined. This could represent an important limitation for basic and translational applications of bioprinted models, including those of the nervous system. Future studies are necessary for identifying more physiological, standardized and defined alternatives to Matrigel. To this direction, promising results have been recently obtained with decellularized extracellular matrix, used for the bioprinting of liver and hearth constructs [9].

Due to the vulnerability of neurons, their viability post printing is a major concern. In this regard, our results (70–80% of live cells) are comparable to previous works using hiPSC-derived neural progenitors [13,14] or an immortalized human neural stem cell line [27]. Moreover, our method allows long term survival of human neurons, up to 70 days post printing. To the best of our knowledge, this is the longest time of maintenance of hiPSC-derived neurons in 3D bioprinted constructs (14 days in [13], 30 days in [14], 40 days in [4] and 41 days in [26]). Further, this work suggests a possible approach to overcome some practical challenges associated with the bioprinting of 3D in vitro models containing cells of limited availability. In order to be able to produce constructs with arbitrary, high cellular density, without affecting the number of samples obtainable from each experiment, we adjusted the amount of bioink necessary for each construct to a few microliters (3 to 5 µL per sample). The dimension, visibility and weight of these samples are very limited, and their handling and maintenance in floating culture condition can be very challenging. To overcome this, we used functionalized micro-slides as receiving substrate during the printing step that guaranteed a prolonged adhesion of the samples to a flat, clear glass surface.

We here report that cortical 3D bioprinted constructs, as well as parallel 2D cultures, display functional properties typical of immature neuronal networks. Indeed, calcium imaging experiments showed sustained calcium spontaneous activity already at DPP7, in line with that reported for 3D bioprinted iPSCs [4,27], and spinal neural progenitors [13], thus suggesting that the printing process does not prevent the development of a functional network. However, passive and active neuronal properties, analyzed at single cell level by means of patch clamp recordings, were typical of immature neurons, and the absence of spontaneous synaptic activity indicated that network activity was mainly not dependent on action potential firing. This result is in line with data on 2D culture at the same time point.

This study opens the possibility for generating more complex human neural 3D constructs, for instance by printing mixed populations with precise ratios of neuronal and glial cells and/or printing iPSCs carrying pathogenic mutations associated to neurological diseases. Notably, the bioprinting approach used herein can be further implemented with more sophisticated microfluidic platforms that might allow the deposition of multiple materials and/or multicellular bioink within a single scaffold, by simultaneously extruding different bioinks or by rapidly switching between one bioink and another, as previously described [16], with the aim of controlling the localization of individual cell types in predetermined positions of the 3D construct. Different specific neuronal subtypes, which can be obtained by iPSC differentiation, might be used as the cellular components of 3D constructs for disease models and drug screening. In the case of complex diseases with clear non-cell autonomous contribution, neural and non-neural cells could be printed together. In the long term, further development of this technology could provide bioprinted cortical neural constructs that can be exploited as customized, standardized and scalable pre-clinical models for drug safety and toxicity studies.

## 5. Conclusions

In this paper we report the generation of a novel type of bioprinted 3D neuronal construct, based on cortical neurons and glial cells derived from hiPSCs. The cortical construct develops molecular, morphological and functional properties of neuronal networks and can be used for future disease modeling studies as well as for drug screening.

## Supplementary Materials

The following are available online at https://www.mdpi.com/2077-0383/8/10/1595/s1, Figure S1: The custom bioprinter used in this work, Figure S2: Neural differentiation of human iPSCs, Figure S3: Effects of the treatment with Alginate-lyase on the bioprinted specimen, Figure S4: Quantitative RT-PCR analysis, Figure S5: Assessment of the 2D conventional cultures functionality, Table S1: Primers used in this study, Video S1 and S2: 3D bioprinting.
