# Supplementary material for: 3D Bioprinted Human Cortical Neural Constructs Derived from Induced Pluripotent Stem Cells
Source: J Clin Med. 2019 Oct 2;8(10):1595. doi: 10.3390/jcm8101595 (PMC6832547; doi:10.3390/jcm8101595)
Supplement: Supplementary file 1 [file jcm-08-01595-s001.zip › Salaris_2019_Supplementary_R1/Salaris_2019_Supplementary_JCM_R1.pdf]

Supplementary Information

3D bioprinted functional human neural constructs derived from induced Pluripotent Stem Cells

Federico Salaris[1,2], Cristina Colosi[1], Carlo Brighi[1], Alessandro Soloperto[1], Valeria de Turris[1], Maria Cristina Benedetti[2], Silvia Ghirga[1], Maria Rosito[1], Silvia Di Angelantonio[1,3,*], Alessandro Rosa[1,2,*]

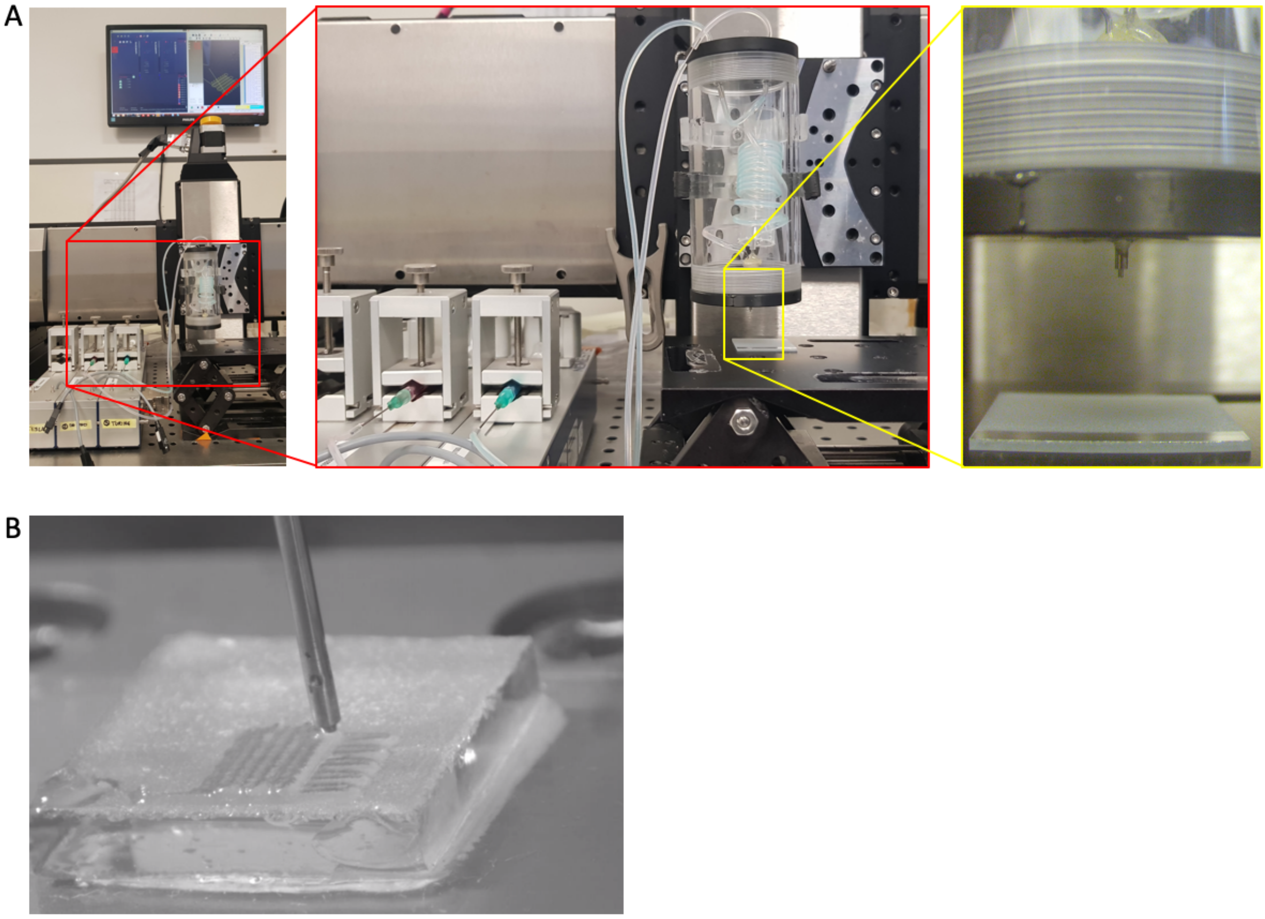

Figure S1. The custom bioprinter used in this work

(A) Photograph of the 3D-bioprinter machine. Close-up pictures show increasing magnifications of the extrusion system. (B) Picture of the construct during the printing process.

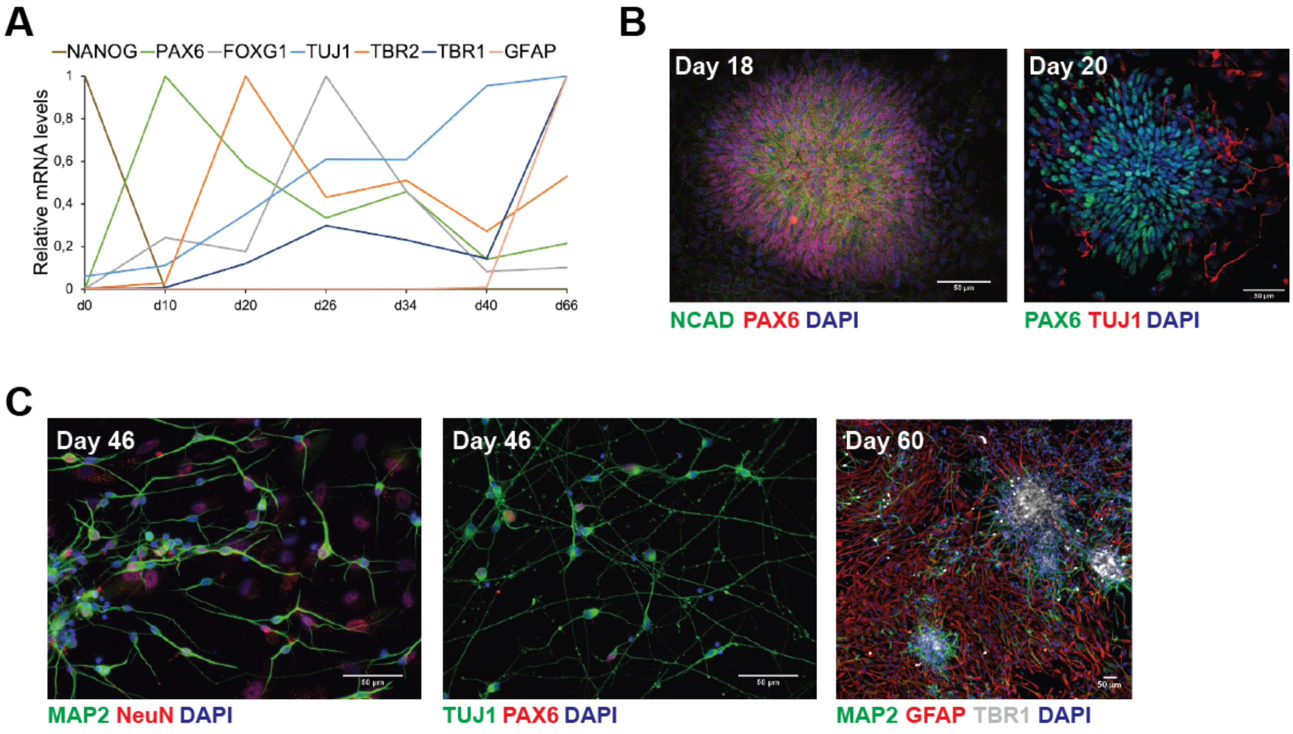

Figure S2. Neural differentiation of human iPSCs

(A) Real-time qRT-PCR analysis of the expression of the indicated markers during iPSC differentiation. Targets were analyzed by real-time qRT-PCR with SYBR Green Power-UP (Thermo Fisher Scientific) in a ViiA 7 Real-Time PCR System (Applied Biosystems). The internal control used was the housekeeping gene ATP5O. Primers sequences are reported in Table S1. (B) Immunostaining showing expression of the indicated neural genes during the NPC formation stage of the differentiation protocol. Antibodies: anti-PAX6 (1:50), anti-NCAD (1:100), anti-TUJ1 (1:1000), anti-MAP2 (1:2000), anti-NeuN (1:50), anti-GFAP (1:500), anti-TBR1 (1:150). (C) Immunostaining showing expression of neuronal and astrocyte markers during the neuronal maturation stage of the differentiation protocol.

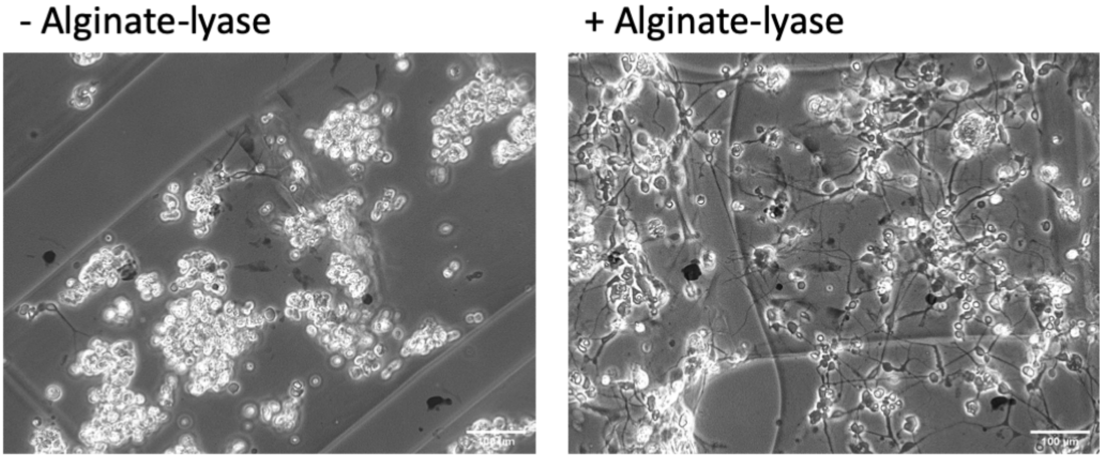

Figure S3. Effects of the treatment with Alginate-lyase on the bioprinted specimen
Photographs of the bioprinted specimen untreated or treated with the enzyme Alginate-lyase. Bioprinted samples were left untreated (left image) or incubated with Alginate-lyase 3 hours after the printing process (right image) and imaged at DPP1.

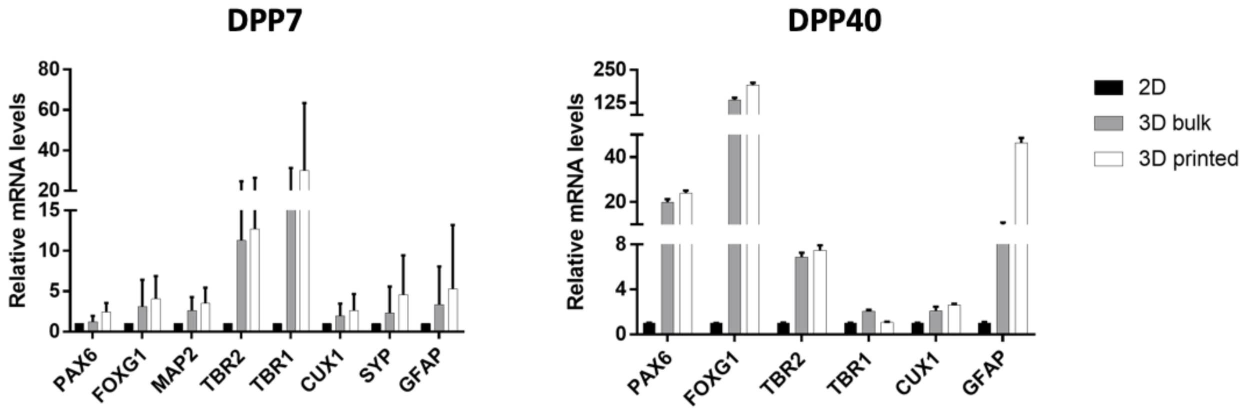

Figure S4. Quantitative RT-PCR analysis

Real-time qRT-PCR analysis of the indicated neuronal progenitors (PAX6, FOXG1, TBR2), neuronal (MAP2, SYP), cortical neurons (TBR1, CUX1) and astrocyte (GFAP) markers on the indicated samples at DPP7 (3 constructs) and DPP40 (1 construct).

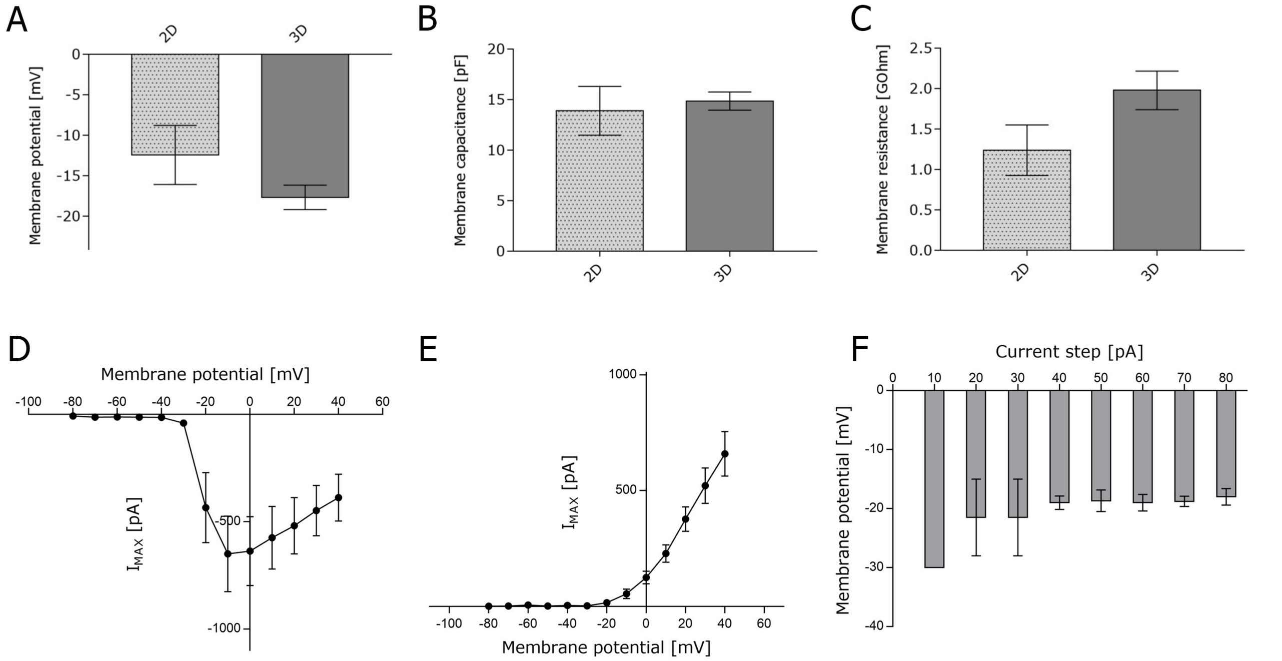

Figure S5. Assessment of the 2D conventional cultures functionality

Bar charts representing: (A) membrane resting potential, (B) cell capacitance and (C) membrane resistance value comparison between 2D conventional cultures and 3D bioprinted constructs. No significant differences were observed between the two culture conditions. I/V plots showing: (D) Average trace of the large inward voltage-dependent Na^+ currents and (E) average trace of the outward voltage-dependent K^+ currents in iPSC-derived cortical neurons after 40 days in vitro in conventional 2D cultures. (F) Bar plot indicating the average threshold membrane potential for each current step required to elicit firing in iPSC-derived cortical neurons after 40 days in vitro in conventional 2D cultures. Data are presented as mean ± s.e.m.

ATP5O FW	ACTCGGGTTTGACCTACAGC
ATP5O RV	GGTACTGAAGCATCGCACCT
CUX1 FW	AGTCCATGGAGTTTGCACCGTC
CUX1 RV	GAGCGGTTCTTCTCCAGCAACA
FOXG1 FW	AGGAGGGCGAGAAGAAGAAC
FOXG1 RV	TCACGAAGCACTTGTTGAGG
GAPDH FW	GGAAGGTGAAGGTCGGAGTC
GAPDH RV	TTACCAGAGTTAAAAGCAGC
GFAP FW	GATCAACTCACCGCCAACAG
GFAP RV	ATAGGCAGCCAGGTTGTTCT
MAP2 FW	TTCCTCCATTCTCCCTCCTCGG
MAP2 RV	TCTTCCCTGCTCTGCGAATTGG
NANOG FW	CCAAATTCTCCTGCCAGTGAC
NANOG RV	CACGTGGTTTCCAAACAAGAAA
PAX6 FW	ATGTGTGAGTAAAATTCTGGGCA
PAX6 RV	GCTTACAACTTCTGGAGTCGCTA
SYP FW	CGGACATGGACGTGGTGAATCA
SYP RV	CACTCTCGGTCTTGTTGGCACA
TBR1 FW	CAACGGAGCCTACAACAGCCTC
TBR1 RV	TGGTAGAACGGAGCTCCTTGGT
TBR2 FW	CTTCTTCCCGGAGCCCTTTGTC
TBR2 RV	TTCGCTCTGTTGGGGTGAAAGG
TUJ1 FW	CCCGGAACCATGGACAGTGT
TUJ1 RV	TGACCCTTGGCCCAGTTGTT

Table S1. Primers used in this study
